# Human IFIT3 Protein Induces Interferon Signaling and Inhibits Adenovirus Immediate Early Gene Expression

**DOI:** 10.1128/mBio.02829-21

**Published:** 2021-11-02

**Authors:** Aniska Chikhalya, Meike Dittmann, Yueting Zheng, Sook-Young Sohn, Charles M. Rice, Patrick Hearing

**Affiliations:** a Department of Microbiology & Immunology, Stony Brook University, New York, New York, USA; b Department of Microbiology, NYU Grossman School of Medicine, New York, New York, USA; c Synthego Corporation, Menlo Park, California, USA; d Laboratory of Virology and Infectious Disease, The Rockefeller University, New York, New York, USA; Princeton University

**Keywords:** IFIT, Adenovirus, IFN, MAVS, STING, IFIT3, adenovirus, interferon

## Abstract

Interferons (IFNs) are one of the hallmarks of host antiviral immunity. IFNs exert their antiviral activities through the induction of IFN-stimulated genes (ISGs) and antiviral proteins; however, the mechanism by which ISGs inhibit adenovirus (Ad) replication is not clearly understood. IFNs repress Ad immediate early gene expression and, consequently, all subsequent aspects of the viral life cycle. In this study, we found that IFN-induced protein with tetratricopeptide repeats 3, IFIT3 (ISG60), restricts Ad replication. IFIT3 repressed Ad E1A immediate early gene expression but did not alter Ad genome entry into the nucleus. Expression of IFIT3 led to phosphorylation of TBK1, IRF3, and STAT1; increased expression of IFNβ and ISGs; and required IFIT1 and IFIT2 partner proteins. During RNA virus infections, it is known that IFIT3 stimulates IFN production through mitochondrial antiviral signaling (MAVS)-mediated activation of TBK1 which synergizes activation of IRF3 and NF-κB. MAVS or TBK1 depletion in cells expressing IFIT3 blocked IFN signaling and reversed the Ad replication restriction. In addition, STING depletion phenocopied the effect suggesting that IFIT3 activates the STING pathway with cross talk to the MAVS pathway. This occurs independently of viral pathogen-associated molecular patterns (PAMPs). These results demonstrate that the expression of a single ISG, IFIT3, activates IFN signaling and establishes a cellular antiviral state independent of viral PAMPs.

## INTRODUCTION

Adenoviruses (Ads) are ubiquitous pathogens that can infect a wide range of vertebrates. Ad primarily cause self-limiting respiratory and gastrointestinal tract infections in humans (1). However, in certain populations like the immunocompromised, elderly, and infants, Ad infection can cause severe disease (2). Ad infections result in a robust immune response, including production of interferons (IFNs), an important class of cytokines released in response to viral infections. Although type I and II IFNs (IFNα/β and IFNγ, respectively) inhibit the replication of divergent human Ads (3), Ad evolved several mechanisms that block IFN signaling and the antiviral activities of certain IFN-stimulated genes (ISGs). Ad E1A proteins bind and sequester STAT transcription factors activated in response to IFN and inhibit the induction of ISGs (4). Ad E1A also binds and disrupts the hBre1 transcription complex and prevents IFN-induced histone monoubiquitination and associated ISG expression (5, 6). Both actions of E1A lead to a global suppression of ISG expression. Analogously, the Ad E1B-55K protein also inhibits ISG expression through its transcriptional repression domain (7, 8). Promyelocytic nuclear bodies (PML-NB) play an important role in cellular intrinsic and IFN-induced antiviral immunity (9). The Ad E4ORF3 protein antagonizes PML-NB functions by disrupting these structures and sequestering antiviral components including PML-NB and Daxx (10, 11). The Ad E1B-55K-E4ORF6 ubiquitin ligase complex also targets Daxx for proteasomal degradation (12). Finally, Ad VA-RNA-I inactivates PKR thereby preventing phosphorylation of the eIF2α translation factor and inhibition of global protein translation during late phase of viral infection (13). The host IFN system has many redundant pathways that likely evolved because viruses evolved these evasion mechanisms.

Despite in-depth knowledge of pathways leading to IFN induction and viral counter mechanisms thereof, the mode of action of individual ISG products to inhibit Ad remain unclear. One specific IFN-mediated inhibitory step is on repression of E1A expression (3), but there are likely other effects on various steps of the viral life cycle. We utilized an established high-throughput image-based screen (14) to determine the individual effects of 401 different ISGs on Ad replication and viral spread. Four ISGs (MAP3K14, RIPK2, TRIM25, and IFIT3) were identified that decreased Ad viral spread in cultured cells. Among these hits, IFIT3 had the greatest inhibitory effect on Ad replication and had not been identified in previous screens of a number of RNA viruses (14). The IFN-induced protein with tetratricopeptide repeats (IFIT) family has been studied extensively in the context of RNA viruses, but little is known about IFIT3 effects on DNA viruses (15–17).

Human IFIT genes IFIT1 (ISG56), IFIT2 (ISG54), IFIT3 (ISG60), and IFIT5 (ISG58) are encoded on chromosome 10. The IFIT proteins are robustly induced by type I IFNs, viral infection, and lipopolysaccharide. They are characterized by unique helix-turn-helix motifs called tetratricopeptide repeats (TPR), which serve as scaffolds to allow protein-protein and protein-RNA interactions (15–17). TPR motifs in various proteins are crucial for functions including protein transport, translation initiation, cell migration, proliferation, antiviral signaling, and viral replication. Recent studies indicate that IFIT proteins play an important role in antiviral processes restricting viral replication, altering protein synthesis, binding to viral RNAs, or interacting with viral structural and nonstructural proteins through direct and indirect mechanisms (15–17). Direct-acting mechanisms include the ability of IFIT1 protein to form a tripartite complex with IFIT2 and IFIT3 at the 5′ end of mRNAs containing a free 5′-triphosphate, thereby blocking translation. IFIT1 also binds to 5′ caps that lack 2’-O-methylation and inhibits translation. Both free 5′ triphosphate groups and 5′ caps that lack 2’-O-methylation are hallmarks of many viral RNAs. In addition, IFIT1 and IFIT2 proteins bind eukaryotic transcription factor 3 (eIF3) and globally block cap-dependent translation. Through an indirect mechanism, IFIT proteins are reported to play a role in antiviral signal pathway transduction and potentiate mitochondrial antiviral signaling (MAVS) activation of TNFR-associated factor family member-associated NF-κB activator-binding kinase (TBK1) (18). TBK1 activates IRF3 and NF-κB to turn on IFNβ gene expression and induce IFN signaling and ISG expression. Considerably less is understood about the role of IFIT proteins in the regulation of DNA virus replication. We investigated the inhibitory effect of IFIT3 on Ad replication and demonstrate that IFIT3 expression induces the expression of IFNβ via the STING/TBK1 pathway to activate IFN signaling. MAVS, IFIT1, and IFIT2 are required for this process which occurs independently of viral PAMPs.

## RESULTS

### A gain-of-function screen reveals several ISGs as inhibitors of Ad spread.

**FIG 1:**
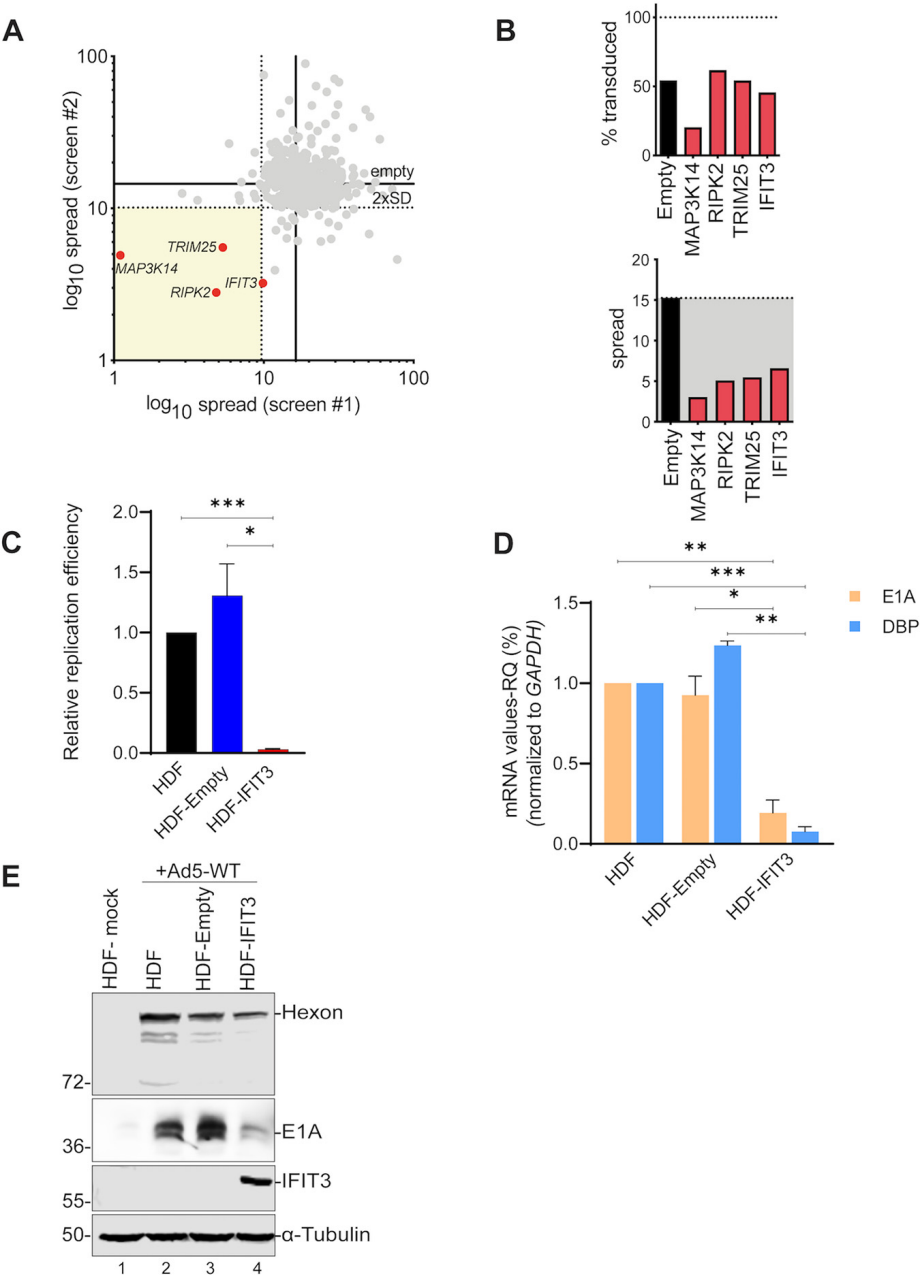
IFIT3 inhibits adenovirus (Ad) replication and early gene expression. (A) Effects of 401 individual (interferon-stimulated genes [ISGs]) on Ad5-enhanced green fluorescent protein (EGFP) spread in A549 cells. ISGs inhibiting more than two standard deviations from the mean in two independent screens are indicated with red dots. Log_10_ spread represents the ratio of infected cells at 60 hpi/24 hpi. (B) The top bar graph shows the percent of A549 cells transduced by Lentivirus-ISG vectors as measured by RFP expression. The bottom bar graph shows confirmation assays of selected ISGs on Ad5 spread in A549 cells in follow up experiments. (C) Wild-type Ad5 (HAdV-C5) replication was analyzed in HDF cells compared to cells transduced with the empty Lentivirus vector or vector expressing IFIT3. Cells were infected with HAdV-C5 at 200 P/cell, harvested at 5 and 48 hpi, and viral DNA replication quantified by quantitative PCR. After normalizing the viral DNA copy numbers to glyceraldehyde-3- phosphate dehydrogenase (GAPDH), the fold-increase in viral copy numbers was calculated by normalizing the amount of DNA present at 48 h to the amount present at 5 h. The data are plotted as mean ± SD, *n* = 3. (*, *P* ≤ 0.05; ***, *P* ≤ 0.001). (D) HDF, HDF-Empty, and HDF-IFIT3 cells were infected with HAdV-C5 at 200 P/cell. Ad early mRNA levels were quantified by quantitative real time PCR with RNA samples isolated 48 hpi. The results were normalized to GAPDH mRNA levels and fold change in HDF-IFIT3 cells was compared to HDF and HDF-Empty cell lines plotted as mean ± SD, *n* = 3. (*, *P* ≤ 0.05; **, *P* ≤ 0.01; ***, *P* ≤ 0.001). (E) HDF, HDF-Empty, and HDF-IFIT3 cells were infected with HAdV-C5 at 200 P/cell. Total cell extracts were harvested at 48 hpi and Ad early (E1A) and late (Hexon) proteins and IFIT3 were analyzed by Western blotting using specific antisera. α-Tubulin is shown as a loading control for the samples. The positions of relevant molecular weight markers are indicated in the left.

To determine whether individual ISG effectors inhibit the Ad life cycle, we used an established high-throughput, image-based screen comprising 401 ISGs (14, 19). This screen is different from others in that it is multi-cycle and thereby able to capture ISG inhibitors of early and late viral life cycle steps. Human lung A549 cells were transduced with lentiviral vectors to express each of the 401 ISGs individually. Forty-eight hours after transduction, cells were infected with Ad5-enhanced green fluorescent protein (EGFP) (HAdV-C5 expressing EGFP) virus at a low multiplicity of infection (MOI) and viral spread ratio was quantified as the number of EGFP-expressing cells at 60 h postinfection (hpi) relative to 24 hpi for each ISG. The screen was performed twice, using independently generated lentivirus libraries (Fig. 1A). Empty lentivirus vector served as a control showing a spread ratio of 15 (Fig. 1B). Four ISGs reduced the Ad5-EGFP spread ratio greater than two standard deviations from the average spread for the empty vector control in two screens (Fig. 1A). RIPK2 and MAP3K14 are protein kinases involved in cell death (20, 21), TRIM25 is a ubiquitin ligase (22) that plays a key role in the RIG-I signaling pathway, and finally IFIT3 is involved in IFN signaling and plays a critical role in conferring immunity against viruses (15–17). RIPK2 and MAP3K14 were identified in previous ISG screens with RNA viruses (14, 19). TRIM25 and IFIT3 were not identified in any previous screen using this ISG library.

Lentivirus transduction was used to establish pools of A549 cells constitutively expressing IFIT3 or TRIM25. We were not able to establish cell lines that expressed RIPK2 or MAP3K14, perhaps due to cytotoxicity, and these ISGs were not pursued further. Replication of HAdV-C5 was reduced 25-fold in IFIT3-expressing cells compared to non-transduced A549 cells and 15-fold compared to A549 cells transduced with the empty lentivirus vector (Fig. S1A in the supplemental material). TRIM25-expressing cells reduced HAdV-C5 replication ∼5–10-fold (Fig. S1C). The effects of IFIT3 and TRIM25 on Ad spread corresponded to a reduction in E1A immediate early protein expression (Fig. S1B and D). Because A549 cells are aneuploid cancer cells, we wished to examine the effect of IFIT3 and TRIM25 on Ad replication in normal human cells. Pools of normal human diploid fibroblasts immortalized by human telomerase (HDF-TERT cells; referred to as HDF cells) (23) were established that stably express IFIT3, designated HDF-IFIT3. HDF cells, as well as A549 cells used in the initial screen, are both able to produce and to respond to IFNs. TRIM25 was not detectably expressed in HDF cells as measured by Western blotting analysis, and so we focused our attention on IFIT3. HDF-IFIT3 cells were infected with HAdV-C5 at a low MOI, and viral DNA replication was quantified at 48 hpi. IFIT3 expression significantly inhibited virus replication (50-fold) compared with empty vector control (HDF-Empty) or parental HDF cell lines (Fig. 1C). We examined viral E1A and DNA binding protein (DBP) mRNA levels in the presence or absence of IFIT3 expression and found that IFIT3 significantly suppressed the expression of these immediate early and early genes, respectively (Fig. 1D). The reduction in E1A mRNA levels correlated with decreased E1A and hexon protein expression (Fig. 1E, lanes 2 and 3 versus lane 4). In conclusion, we identified IFIT3 as an ISG inhibiting Ad spread, immediate early and early gene mRNA levels, and E1A and hexon protein expression.

10.1128/mBio.02829-21.1 FIG S1 IFIT3 and TRIM25 inhibits adenovirus (Ad) replication and early gene expression in A549 cells. (A) Ad5 replication was analyzed in A549 cells compared to cells transduced with empty Lentivirus vector or vector expressing IFIT3 vector. Cells were infected with HAdV-C5 at 5 P/cell, harvested at 5 and 24 hpi, and viral DNA replication was quantified by quantitative PCR. The data are plotted as mean ± SD, *n* = 3 (** *P* ≤ 0.01; *** *P* ≤ 0.001). (B) A549, A549-Empty, A549-IFIT3 cells were infected with HAdV-C5 at 5 P/cell. Cells were harvested at 24 hpi and analyzed by Western blotting for E1A and IFIT3. α-Tubulin is shown as a loading control for the samples. (C) Ad5 replication was analyzed in A549 cells compared to cells transduced with empty Lentivirus vector or vector expressing TRIM25. Viral DNA replication was analyzed as in (A) (* *P* ≤ 0.05). (D) A549, A549-Empty, A549-TRIM25 cells were infected with HAdV-C5 at 5 P/cell. Cells were harvested at 24 hpi and analyzed by Western blotting for E1A and TRIM25. Download FIG S1, TIF file, 2.6 MB. Copyright © 2021 Chikhalya et al. 2021 Chikhalya et al. https://creativecommons.org/licenses/by/4.0/ This content is distributed under the terms of the Creative Commons Attribution 4.0 International license.

### IFIT3 knockout does not restore Ad5 replication in IFN-treated HDF cells.

**FIG 2:**
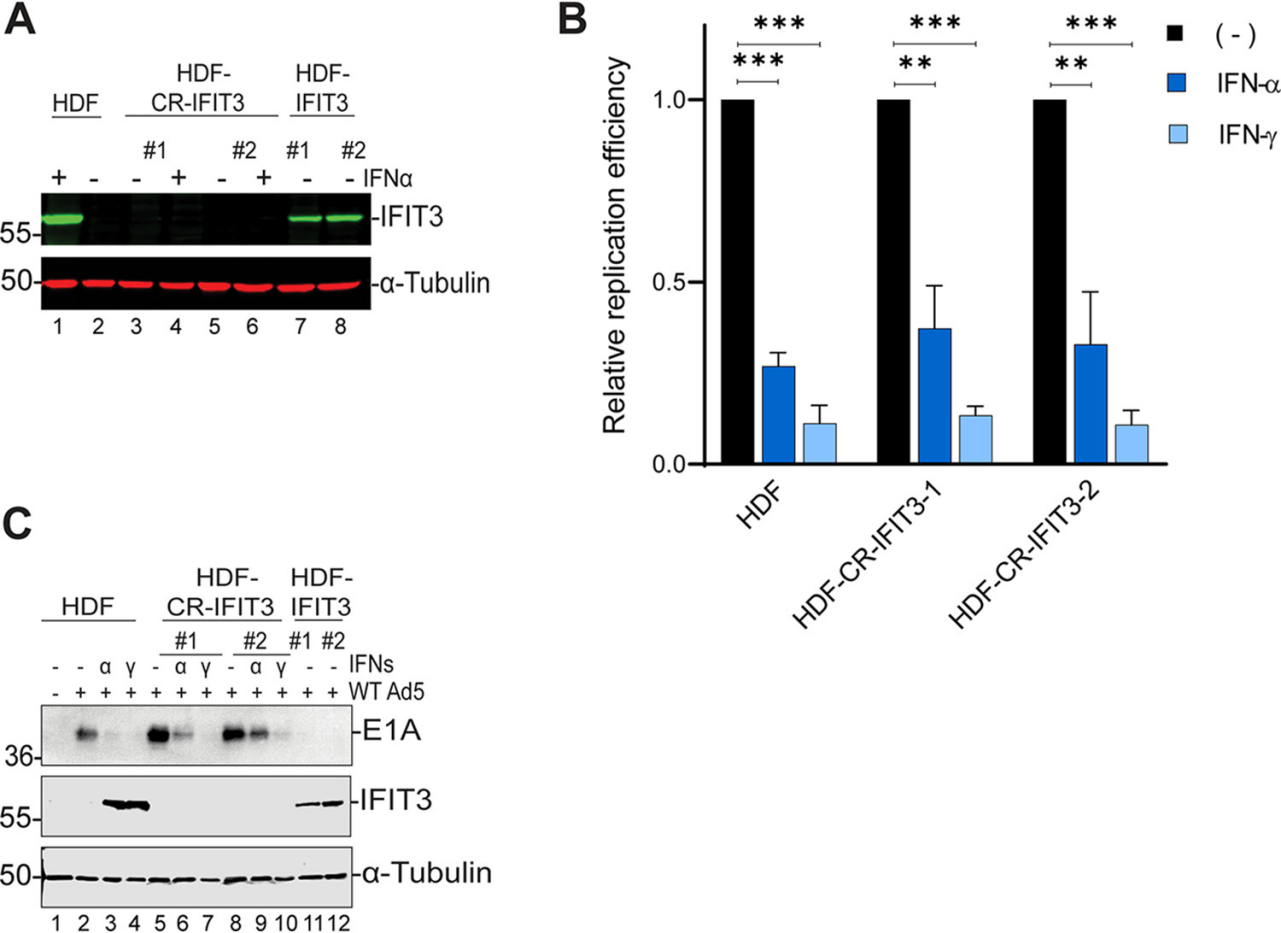
IFIT3 knockout in HDF cells does not affect interferon (IFN)-mediated repression of Ad replication and immediate early gene expression. (A) HDF cells were depleted of IFIT3 using a CRISPR-Cas9 strategy. Cells were either treated with IFNα (1000 U/ml) for 24 h or left untreated to confirm IFIT3 knockout. IFIT3 protein was analyzed by Western blotting. (B) HDF and HDF-IFIT3 knockout cells were treated with IFNα for 24 h or left untreated, and then infected with HAdV-C5 at 200 P/cell. Cells were harvested at 5 and 48 hpi and viral DNA replication was quantified by qPCR. Viral DNA copy numbers were first normalized to glyceraldehyde-3- phosphate dehydrogenase (GAPDH), then the fold-increase in viral copy numbers was calculated by normalizing the amount of DNA present at 48 h to the amount present at 5 h, finally viral copy numbers minus IFN treatment were set at 1 and viral copy numbers plus IFNs were calculated relative to this level. The values are plotted as mean ± SD, *n* = 3. (**, *P* ≤ 0.01; ***, *P* ≤ 0.001). (C) Ad5 E1A and IFIT3 proteins were analyzed by Western blotting.

IFNs α and γ inhibit Ad genome replication by repressing E1A immediate early gene expression (3). To determine whether expression of IFIT3 is integral to the mechanism by which IFNs repress E1A, we generated IFIT3 knockout clones using a lentiviral CRISPR-Cas9 approach (Fig. 2A, lanes 3–6). To evaluate Ad replication in the HDF-IFIT3 knockouts (designated CR-IFIT3), cells were incubated with IFNα, IFNγ, or left untreated for 24 h, followed by HAdV-C5 infection at low MOI. Deletion of IFIT3 in HDF cells did not alter the ability of IFNs to inhibit Ad replication (Fig. 2B). Similarly, the inhibition of E1A immediate early gene expression by IFNs was not affected in CR-IFIT3 cells compared to HDF cells (Fig. 2C, lanes 1–9). Fig. 2C also shows that the HDF cells with ectopic IFIT3 expression used in these experiments expressed IFIT3 at physiologically relevant levels similar to that seen following IFNα or IFNγ induction (lanes 3 and 4 versus lanes 11 and 12). The results of these experiments suggest that IFIT3 is one of the ISGs responsible for E1A suppression.

### IFIT3 does not block nuclear accumulation of Ad genomes.

DNA viruses carry out genome synthesis in the nucleus, and trafficking of viral genomes across the nuclear envelope is required to establish a productive infection (24). The process of Ad entry into the cell and import of the viral genome into the nucleus has been elegantly and extensively studied (25) and many host and viral proteins play a critical role in the ability of a virus to enter a cell and its nucleus (26, 27). IFIT proteins localize in the cytoplasm of various cells (28, 29). Immunofluorescence was used to determine the cellular localization of IFIT3 in parental and HDF-IFIT3 cell types. Parental cells were treated with IFNα as a positive control, and a set of cells were infected with HAdV-C5 to determine if infection affected IFIT3 localization. IFIT3 showed diffuse, total cytoplasmic localization in HDF-IFIT3 and following IFNα treatment (Fig. S2A in the supplemental material). Ad5 infection did not alter IFIT3 localization (Fig. S2B and C).

10.1128/mBio.02829-21.2 FIG S2 IFIT3 localizes to the cytoplasm in uninfected and adenovirus (Ad)5-infected cells. (A) IFIT3 subcellular localization was evaluated by immunofluorescence in untreated HDF cells, HDF cells treated with IFNα for 24 h, and in HDF-IFIT3 cells; IFIT3 (FITC), DAPI and merged images are shown. (B, C) HDF-IFIT3 cells (B) and HDF cells treated with interferon (IFN)α for 24 h (C) were infected with HAdV-C5 at 1000 P/cell and immunostained for IFIT3 (FITC) and Ad DBP (red), and stained with DAPI, at 48 hpi. Download FIG S2, TIF file, 2.2 MB. Copyright © 2021 Chikhalya et al. 2021 Chikhalya et al. https://creativecommons.org/licenses/by/4.0/ This content is distributed under the terms of the Creative Commons Attribution 4.0 International license.

**FIG 3:**
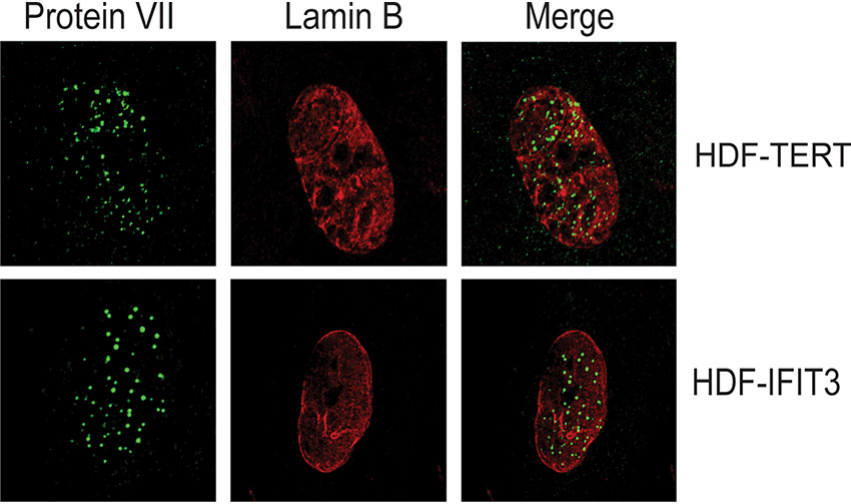
IFIT3 does not block nuclear accumulation of Ad genomes. HDF and HDF-IFIT3 cells were infected with HAdV-C5 at 1000 P/cell and immunostained for Ad core Protein VII (FITC) and a nuclear protein, Lamin B (TRITC) at 6 hpi. Merged images are shown on the right.

We sought to determine if expression of IFIT3 in HDF cells affected viral genome entry into the nucleus. Ad genome localization in infected HDF and HDF-IFIT3 cells was examined by high-resolution microscopy by visualizing Ad protein VII, a viral core protein associated with the genome through the early phase of infection (30), as a surrogate. There were similar nuclear protein VII levels in both cell types, thus IFIT3 did not block the entry of viral genomes into the nucleus of infected cells (Fig. 3). We thus conclude that IFT3 does not exert its anti-Ad function directly by blocking viral nuclear entry.

### IFIT3 expression activates IFN signaling.

**FIG 4:**
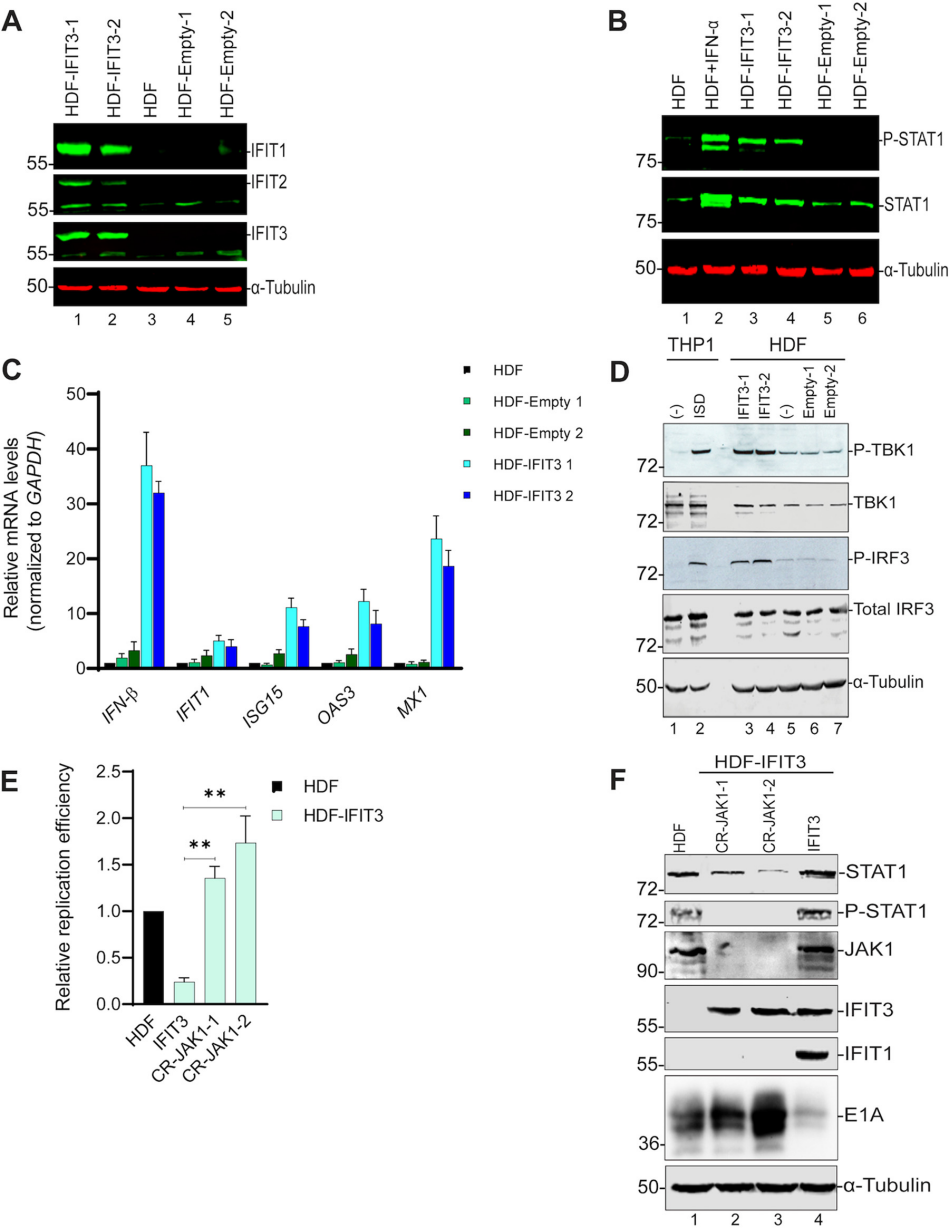
Induction of interferon (IFN) signaling by IFIT3. (A, B) HDF, HDF-Empty and HDF-IFIT3 cell extracts were analyzed by Western blotting using antibodies against (A) IFIT1, IFIT2 and IFIT3, (B) total STAT1 and phosho-STAT1 (Y701). The bands near the bottom of the gel in (A), IFIT2 and IFIT3, are nonspecific, cross-reactive proteins. (C) RNA was isolated from HDF, HDF-Empty and HDF-IFIT3 cells and IFNβ and specific interferon-stimulated genes (ISGs) (IFIT1, ISG15, OAS3, and MX1) mRNA levels were quantified by RT-qPCR. The results were normalized to glyceraldehyde-3- phosphate dehydrogenase (GAPDH) mRNA levels and fold change in HDF-IFIT3 cells was compared to HDF and HDF-Empty cell lines, and plotted as mean ± SD, *n* = 3. (D) HDF, HDF-Empty and HDF-IFIT3 cell extracts were analyzed by Western blotting for total TBK1, phospho-TBK1 (S172), total IRF3, and phopsho-IRF3 (S396). Human THP1 monocytes were left untreated or treated with IFN-stimulatory DNA (ISD) as a positive control for activation of TBK1 signaling lanes 1 and 2. (E) HDF, HDF-IFIT3, and HDF-IFIT3+JAK1 knockout cells were infected with HAdV-C5 at 200 P/cell and viral DNA replication was quantified by qPCR at 48 hpi, as described for Fig. 1C. The data are plotted as mean ± sd, *n* = 3. (**, *P* ≤ 0.01). (F) HDF, HDF-IFIT3, and HDF-IFIT3+JAK1 knockout cells were infected with HAdV-C5 at 200 P/cell and protein expression analyzed by Western blotting at 48 hpi using antibodies against total STAT1, phopsho-STAT1, JAK1, IFIT3, IFIT1, and Ad5 E1A.

During RNA virus infections, IFIT3 stimulates IFN production through MAVS-mediated activation of TBK1, which promotes activation of IRF3 and NF-κB (18). We next sought to determine whether IFIT3 exerts its anti-Ad activity indirectly, by triggering the production of IFNs. HDF, HDF-Empty, and HDF-IFIT3 cells were examined to determine if IFIT3 expression led to the induction of IFN signaling and ISG expression. Western blot analysis demonstrated that IFIT1 and IFIT2 protein expression was elevated in cells that express IFIT3 (Fig. 4A, lanes 1 and 2). IFIT3 expression also induced tyrosine phosphorylation of STAT1 (Fig. 4B, lanes 3 and 4, and Fig. S3B in the supplemental material, lanes 4 and 5). The degree to which IFIT3 inhibited Ad replication and elevated the level of tyrosine-phosphorylated STAT1 was similar to that obtained with treatment of HDF cells with 50 or 500 units of IFNα (Fig. S3A and B). IFIT3 expression also increased the expression of IFNβ and the ISGs IFIT1, ISG15, OAS3 and MX1 as determined by RT-qPCR (Fig. 4C). It has been reported that IFIT3 potentiates antiviral signaling by bridging MAVS and TBK1, in turn inducing IRF3 phosphorylation and IFNβ expression (18). We found that IFIT3-expressing cells had elevated levels of TBK1 phosphorylation and IRF3 phosphorylation compared to the control cells (Fig. 4D, lanes 3 and 4 versus lanes 5–7). Collectively these results indicate that IFIT3 expression in HDF cells activates TBK1 and IRF3, leading to IFNβ production, activation of STAT1, and induction of ISG expression. Notably, activation of this IFN pathway is independent of viral infection.

10.1128/mBio.02829-21.3 FIG S3 Inhibition of adenovirus (Ad) replication by IFIT3 is similar to cells treated with IFNα. (A) Ad5 replication was analyzed in HDF cells treated with interferon (IFN)α and compared to HDF-IFIT3 cells. Cells were pretreated with 500 or 50 U/ml IFNα for 24h, or left untreated, and infected with HAdV-C5 at 200 P/cell. Cells were harvested at 5 and 48 hpi and viral DNA replication was quantified by qPCR. The data are plotted as mean ± sd, *n* = 3 (**, *P* ≤ 0.01; ***, *P* ≤ 0.001). (B) Infected cells as described in (A), were analyzed at 48 hpi by Western blotting for activation of IFN signaling (STAT1, phospho-STAT1 and IFIT3) and Ad5 E1A expression. Download FIG S3, TIF file, 2.2 MB. Copyright © 2021 Chikhalya et al. 2021 Chikhalya et al. https://creativecommons.org/licenses/by/4.0/ This content is distributed under the terms of the Creative Commons Attribution 4.0 International license.

IFN signaling in normal human cells such as HDF results in the inhibition of both Ad replication and viral early and late gene expression (3). To determine if the suppression of Ad replication by IFIT3 was due to IFN signaling, we generated JAK1 knockout cells to block phosphorylation of STAT1, and thus IFN signaling. JAK1 knockouts were generated by a CRISPR-Cas9 strategy in both HDF and HDF-IFIT3 cells. The depletion of JAK1 did not reduce IFIT3 expression and restored Ad replication (Fig. 4E) and E1A expression in infected HDF-IFIT3 cells (Fig. 4F, lanes 2 and 3 versus lane 4) but did not affect Ad replication and E1A expression in the control HDF cells (Fig. S4A and B). These results show that the ability of IFIT3-expressing cells to inhibit Ad is dependent on JAK1.

10.1128/mBio.02829-21.4 FIG S4 JAK1, IFIT2 and IFIT1 knockout in HDF cells does not affect Ad replication and early gene expression. (A) HDF and HDF+JAK1 knockout cells were infected with HAdV-C5 at 200 P/cell and viral DNA replication quantified by qPCR at 48 hpi. The data are plotted as mean ± SEM, *n* = 3. (ns = not statistically significant). (B) HDF and HDF JAK1 knockout cells were infected with HAdV-C5 at 200 P/cell and protein expression analyzed by Western blotting at 48 hpi using antibodies against E1A and JAK1. (C) HDF and HDF+IFIT1 knockout cells were infected with HAdV-C5 at 200 P/cell and viral DNA replication quantified by qPCR at 48 hpi. The data are plotted as mean ± SEM, *n* = 3. (D) HDF and HDF+IFIT1 knockout cells were infected with HAdV-C5 at 200 P/cell and protein expression analyzed by Western blotting at 48 hpi using antibodies against E1A and IFIT1. (E) HDF and HDF+IFIT2 knockout cells were infected with HAdV-C5 at 200 P/cell and viral DNA replication quantified by qPCR at 48 hpi. The data are plotted as mean ± SEM, *n* = 3. (F) HDF and HDF+IFIT2 knockout cells were infected with HAdV-C5 at 200 P/cell and protein expression analyzed by Western blotting at 48 hpi using antibodies against E1A and IFIT2. The asterisk in the IFIT2 blot indicates IFIT3 which cross-reacts with the IFIT2 antibody. Download FIG S4, TIF file, 2.3 MB. Copyright © 2021 Chikhalya et al. 2021 Chikhalya et al. https://creativecommons.org/licenses/by/4.0/ This content is distributed under the terms of the Creative Commons Attribution 4.0 International license.

### TBK1 and STING knockout blocks IFIT3 induction of IFN signaling and the inhibition of Ad replication.

**FIG 5:**
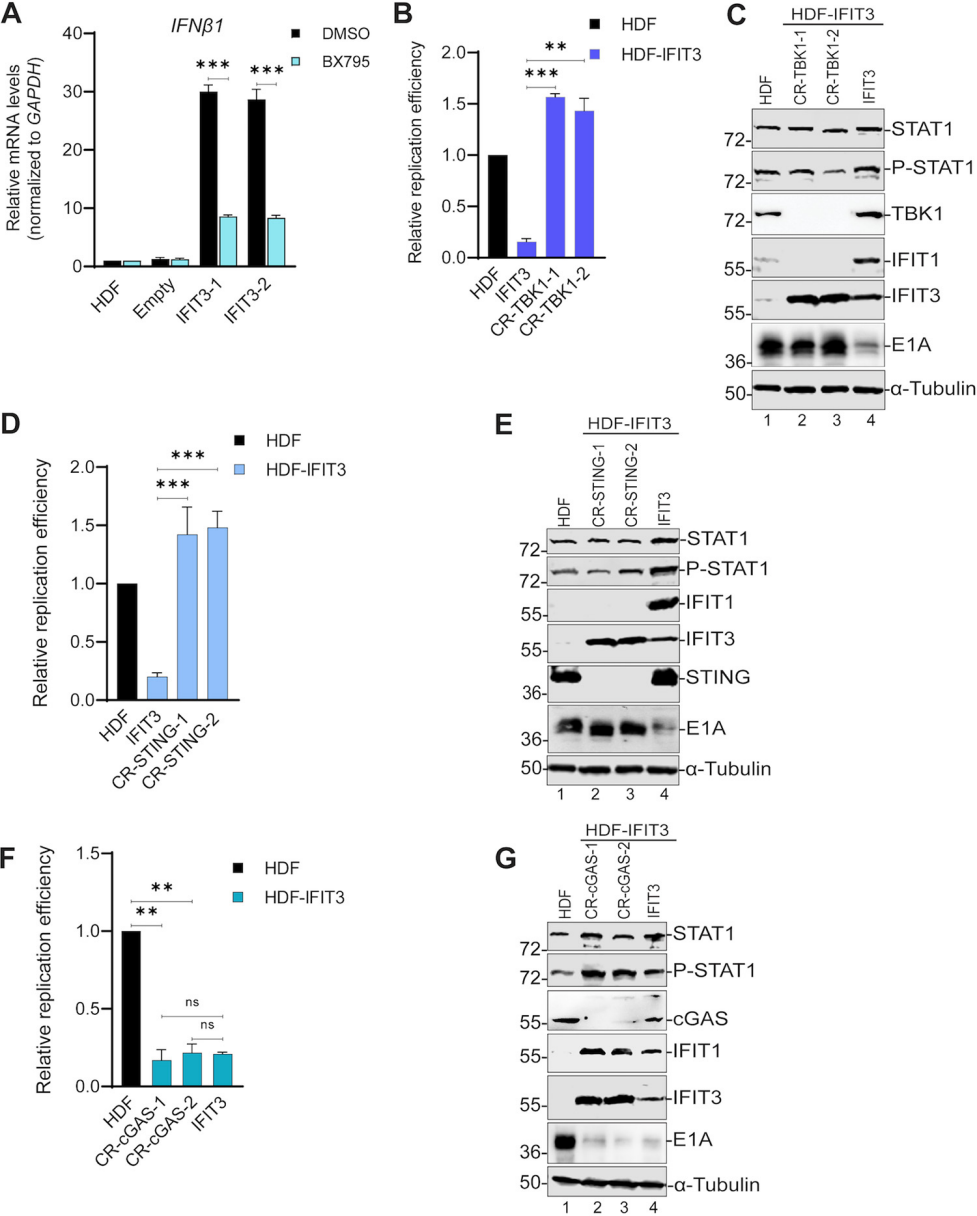
TBK1 and STING knockout blocks IFIT3 induction of interferon (IFN) signaling and the inhibition of adenovirus (Ad) replication. (A) HDF, HDF-Empty and HDF-IFIT3 cells were left untreated or treated with the TBK1 inhibitor BX795 (2 μM) for 24 h. IFN-β mRNA levels were quantified by quantitative real time PCR and normalized to glyceraldehyde-3-phosphate dehydrogenase (GAPDH) mRNA levels. The data are plotted as mean ± SD, *n* = 3. (***, *P* ≤ 0.001). (B) HDF, HDF-IFIT3 (IFIT3), and HDF-IFIT3+TBK1 knockout cells (CR-TBK1) were infected with HAdV-C5 at 200 P/cell and viral DNA replication was quantified by qPCR at 48 hpi as described for Fig. 1C. The data are plotted as mean ± SD, *n* = 3. (**, *P* ≤ 0.01; ***, *P* ≤ 0.001). (C) HDF, HDF-IFIT3 (IFIT3), and HDF-IFIT3+TBK1 knockout cells (CR-TBK1) were infected with HAdV-C5 at 200 P/cell and protein expression analyzed by Western blotting at 48 hpi using antibodies against total STAT1, phopsho-STAT1, TBK1, IFIT1, IFIT3, and Ad5 E1A. (D) HDF, HDF-IFIT3 (IFIT3), and HDF-IFIT3+STING knockout cells (CR-STING) were infected with HAdV-C5 at 200 P/cell and viral DNA replication was quantified by qPCR at 48 hpi as described for Fig. 1C. The data are plotted as mean ± SD, *n* = 3. (***, *P* ≤ 0.001). (E) HDF, HDF-IFIT3 (IFIT3), and HDF-IFIT3+STING knockout cells (CR-STING) were infected with HAdV-C5 at 200 P/cell and protein expression was analyzed by Western blotting at 48 hpi using antibodies against total STAT1, phospho-STAT1, IFIT1, IFIT3, STING, and Ad5 E1A. (F) HDF, HDF-IFIT3 (IFIT3), and HDF-IFIT3+cyclic GMP-AMP synthase (cGAS) knockout cells (CR-cGAS) were infected with HAdV-C5 at 200 P/cell and viral DNA replication was quantified by qPCR at 48 hpi as described for Fig. 1C. The data are plotted as mean ± SD, *n* = 3. (**, *P* ≤ 0.01). (G) HDF, HDF-IFIT3 (IFIT3), and HDF-IFIT3+cGAS knockout cells (IFIT3) were infected with HAdV-C5 at 200 P/cell and protein expression analyzed by Western blotting at 48 hpi using antibodies against total STAT1, phospho-STAT1, IFIT1, IFIT3, cGAS, and Ad5 E1A.

IFIT3 expression in HDF cells leads to elevated levels of TBK1 phosphorylation. We asked if TBK1 was required for the ability of IFIT3 expression to increase IFN signaling and inhibit Ad replication. HDF and HDF-IFIT3 cells were treated with the TBK1 inhibitor BX795 or DMSO for 24 h. A significant decrease in IFNβ expression was observed in HDF-IFIT3 cells treated with BX795 (Fig. 5A). We also generated TBK1 knockout cell lines in the HDF and HDF-IFIT3 backgrounds using CRISPR-Cas9 to determine the effect on Ad5 DNA replication and early gene expression. TBK1 depletion in HDF-IFIT3 cells did not reduce IFIT3 expression (Fig. 5C lanes 2–4) but did restore Ad5 DNA replication (Fig. 5B) and E1A expression (Fig. 5C, lanes 2 and 3 versus lane 4) and had no effect in control cells (Fig. S5A and B).

10.1128/mBio.02829-21.5 FIG S5 TBK1, STING and cGAS knockout in HDF cells does not affect adenovirus (Ad) replication and early gene expression. (A) HDF and HDF+TBK1 knockout cells were infected with HAdV-C5 at 200 P/cell and viral DNA replication quantified by qPCR at 48 hpi, as described for Fig. 1C. The data are plotted as mean ± SEM, *n* = 3. (ns = not statistically significant). (B) HDF and HDF TBK1 knockout cells were infected with HAdV-C5 at 200 P/cell and protein expression analyzed by Western blotting at 48 hpi using antibodies against E1A and total TBK1. (C) HDF and HDF+STING knockout cells were infected with HAdV-C5 at 200 P/cell and viral DNA replication quantified by qPCR at 48 hpi. The data are plotted as mean ± SEM, *n* = 3. (D) HDF and HDF+STING knockout cells were infected with HAdV-C5 at 200 P/cell and protein expression analyzed by Western blotting at 48 hpi using antibodies against E1A and total STING. (E) HDF and HDF+cGAS knockout cells were infected with HAdV-C5 at 200 P/cell and viral DNA replication quantified by qPCR at 48 hpi. The data are plotted as mean ± SEM, *n* = 3. (F) HDF and HDF+cGAS knockout cells were infected with HAdV-C5 at 200 P/cell and protein expression analyzed by Western blotting at 48 hpi using antibodies against E1A and total cGAS. Download FIG S5, TIF file, 2.7 MB. Copyright © 2021 Chikhalya et al. 2021 Chikhalya et al. https://creativecommons.org/licenses/by/4.0/ This content is distributed under the terms of the Creative Commons Attribution 4.0 International license.

TBK1 is a protein kinase involved in many signaling pathways including the cyclic GMP-AMP synthase (cGAS)-STING pathway that is activated in response to viral DNA. Early studies with Ad implicated cGAS as a significant pattern recognition receptor that contributes to anti-Ad responses (4, 31). We wanted to determine if the cGAS-STING pathway was key for the inhibition of Ad replication by IFIT3. We generated STING knockout cell lines using CRISPR-Cas9 in HDF and HDF-IFIT3 cells. Ad DNA replication was not affected in STING-depleted HDF cells (Fig. S5C and D). In contrast, STING depletion in HDF-IFIT3 cells restored Ad replication (Fig. 5D) and E1A gene expression (Fig. 5E, lanes 2 and 3 versus lane 4). STING depletion in HDF-IFIT3 cells did not reduce IFIT3 expression (Fig. 5E, lanes 2–4). These results implicate STING and TBK1 in IFIT3-mediated activation of IFN signaling and the suppression of Ad replication and early gene expression. cGAS binds cytosolic DNA and triggers STING activation via the production of cGAMP (32). To determine if IFIT3 plays a role upstream or downstream of cGAS, we generated cGAS knockout cell lines using CRISPR-Cas9 in HDF and HDF-IFIT3 cells. Ad DNA replication and E1A expression were not significantly affected in cGAS-depleted HDF cells (Fig. S5E and F). Depletion of cGAS did not reduce IFIT3 expression and was unable to restore Ad5 replication (Fig. 5F) or E1A gene expression (Fig. 5G, lanes 2–4) in HDF-IFIT3 cells. These results suggest IFIT3 plays a role upstream of TBK1 but downstream of cGAS.

### MAVS knockout blocks the inhibitory effects of IFIT3 on Ad DNA replication and early gene expression.

**FIG 6:**
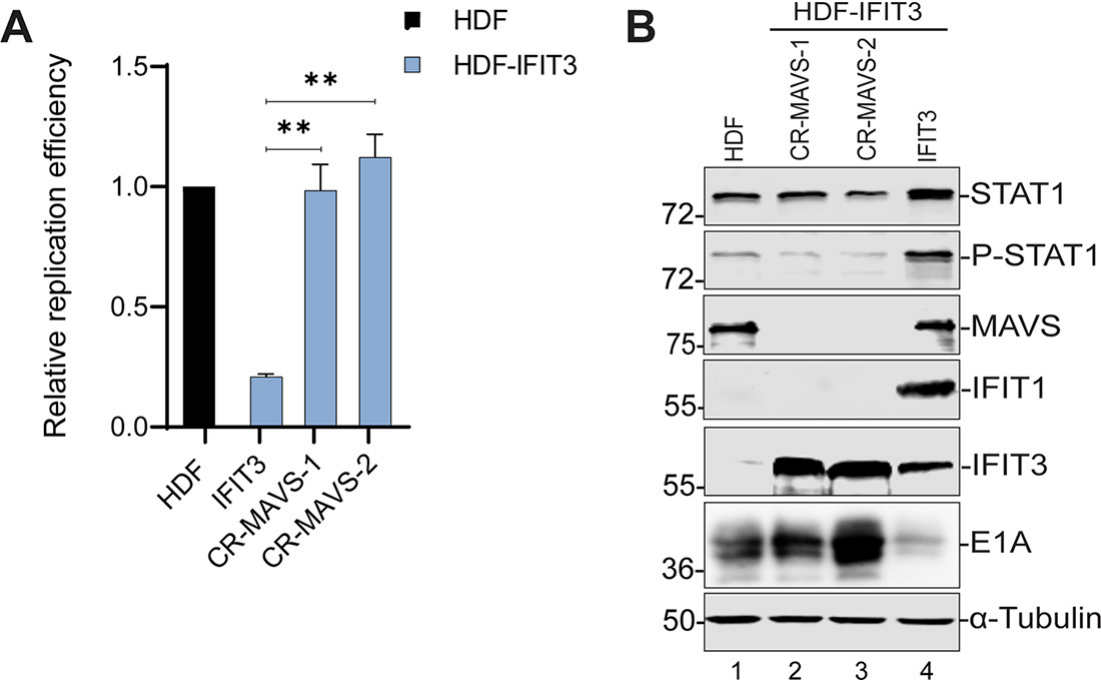
Mitochondrial antiviral signaling (MAVS) knockout blocks the effect of IFIT3 on adenovirus (Ad) replication and early gene expression. (A) HDF, HDF-IFIT3 (IFIT3), and HDF-IFIT3+MAVS (CR-MAVS) knockout cells (CR-MAVS) were infected with HAdV-C5 at 200 P/cell and viral DNA replication was quantified by qPCR at 48 hpi as described for Fig. 1C. The data are plotted as mean ± sd, *n* = 3. (**, *P* ≤ 0.01). (B) HDF, HDF-IFIT3 (IFIT3), and HDF-IFIT3+MAVS knockout cells (CR-MAVS) were infected with HAdV-C5 at 200 P/cell and cell lysates were analyzed by Western blotting at 48 hpi using antibodies against STAT1, phospho-STAT1, MAVS, IFIT1, IFIT3, and Ad5 E1A.

TBK1 is involved in many different signaling pathways including the retinoic acid inducible gene I (RIG-I)/mitochondrial antiviral signaling (MAVS) pathway typically associated with sensing of RNA viruses (33). The IFIT3 protein recruits TBK1 to the MAVS complex on mitochondria allowing for activation of the MAVS signaling pathway (18). We asked if the MAVS pathway was also involved in the effect of IFIT3 on IFN signaling and Ad replication by generating MAVS knockout cell lines using CRISPR-Cas9 in the HDF and HDF-IFIT3 cells. Ad DNA replication and E1A gene expression were not affected in MAVS-depleted HDF cells (Fig. S6A and B). In contrast, MAVS depletion in HDF-IFIT3 cells (CR-MAVS) restored Ad replication (Fig. 6A) and E1A gene expression (Fig. 6B, lanes 2 and 3 versus 4). MAVS depletion in HDF-IFIT3 cells did not reduce IFIT3 expression (Fig. 6B, lanes 2–4).

10.1128/mBio.02829-21.6 FIG S6 Mitochondrial antiviral signaling (MAVS) knockout in HDF cells does not affect adenovirus (Ad) replication and early gene expression. (A) HDF and HDF+MAVS knockout cells were infected with HAdV-C5 at 200 P/cell and viral DNA replication quantified by qPCR at 48 hpi. The data are plotted as mean ± SEM, *n* = 3. (ns = not statistically significant). (B) HDF and HDF+MAVS knockout cells were infected with HAdV-C5 at 200 P/cell and protein expression analyzed by Western blotting at 48 hpi using antibodies against Ad5 E1A and MAVS. (C) Cell extracts from HDF, HDF+MAVS knockout cells, with or without IFIT3 expression, and HDF+STING knockout cells, with or without IFIT3 expression, were analyzed by Western blotting using antibodies against MAVS, STING, and IFIT3. Download FIG S6, TIF file, 2.6 MB. Copyright © 2021 Chikhalya et al. 2021 Chikhalya et al. https://creativecommons.org/licenses/by/4.0/ This content is distributed under the terms of the Creative Commons Attribution 4.0 International license.

We did not anticipate that depletion of both STING and MAVS would block IFIT3 activity in these assays because these effectors are involved in distinct signaling pathways (cGAS and RIG-I, respectively) (34). We analyzed expression of these proteins in the corresponding knockout cell lines. Ablation of MAVS expression did not affect STING expression (Fig. S6C, lanes 2–5), and likewise, ablation of STING expression did not affect MAVS expression (Fig. S6C, lanes 7–10). Thus, the restoration of Ad replication and E1A gene expression observed following STING and MAVS knockout (Fig. 5D and E and 6A and B, respectively) is not due to either of these proteins affecting the expression of the other one. Collectively, these results implicate MAVS, STING and TBK1 in IFIT3-mediated activation of IFN signaling and the suppression of Ad5 replication and E1A immediate early gene expression.

### IFIT1 and IFIT2 expression do not suppress Ad replication.

**FIG 7:**
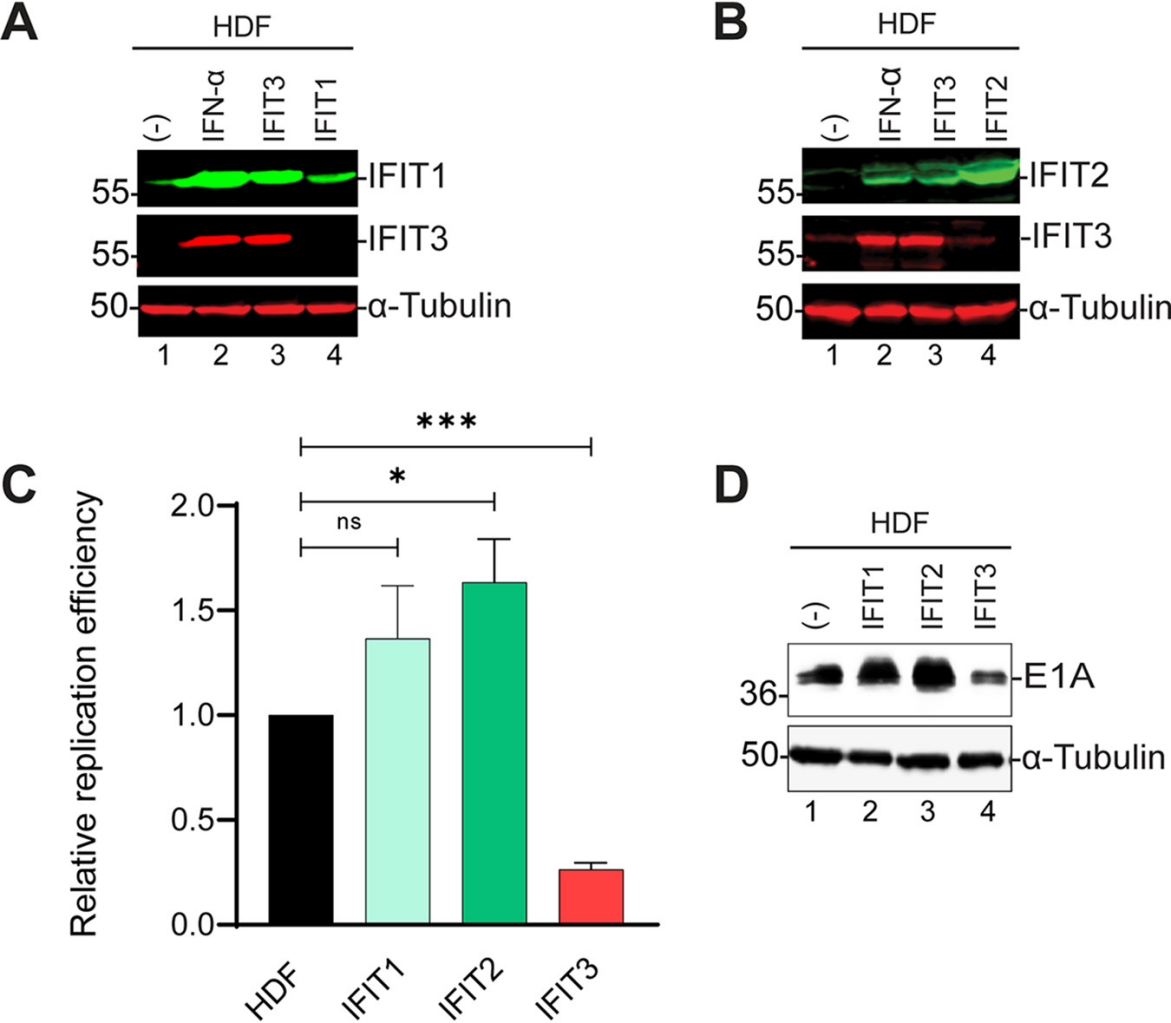
IFIT1 and IFIT2 expression do not inhibit adenovirus (Ad) replication and early gene expression. (A, B) Cell extracts from HDF cells and HDF cells expressing IFIT1, IFIT2, or IFIT3 were analyzed by Western blotting using antibodies against IFIT1, IFIT2 and IFIT3. HDF cells were treated with interferon (IFN)α for 24 h as a positive control. (C) HDF cells and HDF cells expressing IFIT1, IFIT2, or IFIT3 were infected with HAdV-C5 at 200 P/cell and viral DNA replication was quantified by qPCR at 48 hpi as described for Fig. 1C. The data are plotted as mean ± SD, *n* = 3. (*, *P* ≤ 0.05; ***, *P* ≤ 0.001; ns = not statistically significant). (D) HDF cells and HDF cells expressing IFIT1, IFIT2, or IFIT3 were infected with HAdV-C5 at 200 P/cell and cell lysates were analyzed by Western blotting at 48 hpi using antibodies against Ad5 E1A.

Because both IFIT1 and IFIT2 were upregulated by IFIT3 expression (Fig. 4A), and it is known that these IFIT proteins form functional complexes (34, 35), we asked if IFIT1 or IFIT2 expression alone inhibited Ad replication. HDF cells expressing IFIT1 or IFIT2 were generated (Fig. 7A and B) and Ad replication was analyzed. IFIT3 expression induced the expression of both IFIT1 and IFIT2 (Fig. 7A and B, lanes 1 versus 3), as previously observed (Fig. 4A). Neither IFIT1 nor IFIT2 suppressed Ad replication, in contrast to IFIT3 (Fig. 7C). Early gene expression also was not affected by the expression of IFIT1 or IFIT2 compared to IFIT3 (Fig. 7D, lanes 2 and 3 versus lane 4).

### IFIT1 and IFIT2 are required for IFIT3 inhibition of Ad replication and immediate early gene expression.

**FIG 8:**
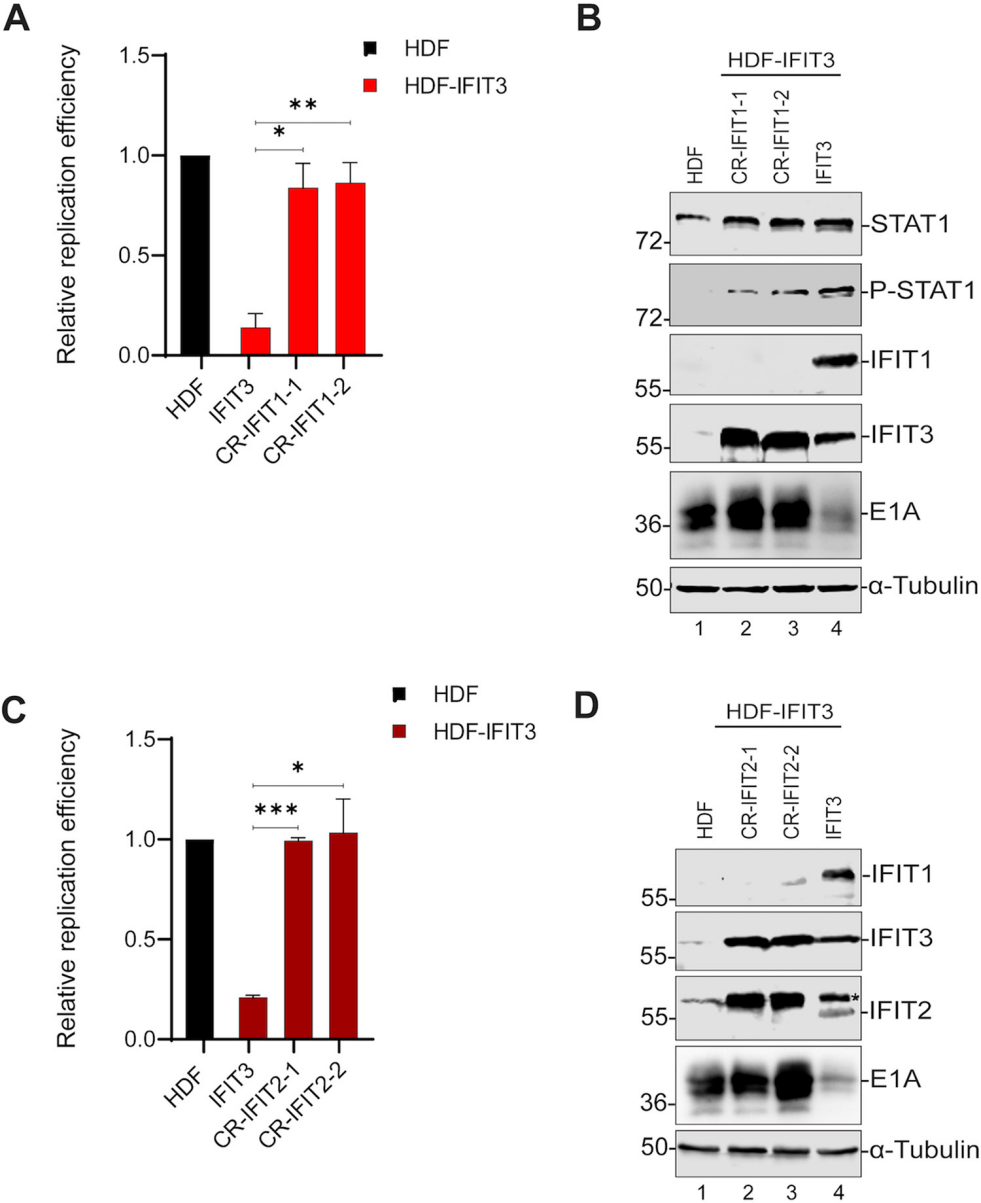
IFIT1 is required for IFIT3 inhibition of Ad replication and early gene expression. (A) HDF-IFIT3 cells were depleted of IFIT1 using CRISPR-Cas9. HDF, HDF-IFIT3 (IFIT3), and HDF-IFIT3+IFIT1 knockout cells (CR-IFIT1) were infected with HAdV-C5 at 200 P/cell and viral DNA replication was quantified using qPCR at 48 hpi as described for Fig. 1C. The data are plotted as mean ± SD, *n* = 3. (*, *P* ≤ 0.05; **, *P* ≤ 0.01). (B) Cell HDF, HDF-IFIT3 (IFIT3), and HDF-IFIT3+IFIT1 knockout cells (CR-IFIT1) were infected with HAdV-C5 at 200 P/cell and cell lysates were analyzed by Western blotting at 48 hpi using antibodies against total STAT1, phospho-STAT1, IFIT1, IFIT3, and Ad5 E1A. (C) HDF-IFIT3 cells were depleted of IFIT2 using CRISPR-Cas9. HDF, HDF-IFIT3 (IFIT3), and HDF-IFIT3+IFIT2 knockout cells (CR-IFIT2) were infected with HAdV-C5 at 200 P/cell and viral DNA replication was quantified using quantitative PCR at 48 hpi as described for Fig. 1C. The data are plotted as mean ± SD, *n* = 3. (*, *P* ≤ 0.05; ***, *P* ≤ 0.001). (D) HDF, HDF-IFIT3 (IFIT3), and HDF-IFIT3+IFIT2 knockout cells (CR-IFIT2) were infected with HAdV-C5 at 200 P/cell and cell lysates were analyzed by Western blotting at 48 hpi using antibodies against IFIT2, IFIT3, and Ad5 E1A. The asterisk in the IFIT2 blot indicates IFIT3 which cross-reacts with the IFIT2 antibody.

Many studies have shown that IFIT proteins interact with one another to promote their antiviral activity (34, 35). Since IFIT1 was robustly expressed in HDF-IFIT3 cells but not expressed in the HDF-IFIT3 TBK1, STING, and MAVS knockout cells (Fig. 5C and E and 6B, lane 4), we wanted to examine if IFIT1 is required for IFIT3 activity. We ablated IFIT1 expression in HDF and HDF-IFIT3 cells using CRISPR-Cas9 and compared Ad replication to HDF-IFIT3-infected cells. IFIT1 knockout in HDF cells had no effect on Ad5 replication and early gene expression (Fig. S4C and D). IFIT1 knockout in HDF-IFIT3 cells restored Ad DNA replication (Fig. 8A) and E1A gene expression (Fig. 8B, lanes 2 and 3 versus 4). We also tested if IFIT2 was required for IFIT3 activity. We ablated IFIT2 expression in HDF and HDF-IFIT3 cells using CRISPR-Cas9 and compared Ad replication to HDF-IFIT3-infected cells. IFIT2 knockout in HDF cells had no effect on Ad replication and early gene expression (Fig. S4E and F). IFIT2 knockout in HDF-IFIT3 cells restored Ad DNA replication (Fig. 8C) and E1A gene expression (Fig. 8D, lanes 2 and 3 versus 4). These results demonstrate that both IFIT1 and IFIT2 are required for IFIT3 activity.

## DISCUSSION

Ad has been used extensively in different oncolytic therapeutic approaches as well as for vaccine delivery. Understanding the host innate immune responses to Ad infection will improve the quality of vectors being designed for translational research. Using an image based high-throughput microscopy screen, we identified four ISGs that limited Ad5-EGFP spread in human lung A549 cells (Fig. 1; MAP3K14, RIPK2, TRIM25, and IFIT3). MAP3K14 and RIPK2 were identified in previous screens using a number of different RNA viruses and are nonspecific inhibitors; TRIM25 and IFIT3 were uniquely identified in this screen. We were surprised that such a limited number of ISGs were identified as hits in the Ad5-EGFP screen since numerous ISGs were found to inhibit the replication and spread of many different RNA viruses and retroviruses (14, 19, 36–38). This may reflect, in part, the inhibition of ISG activities by different Ad IFN antagonists as well as unique aspects of the Ad life cycle. For example, Ad is a non-enveloped virus that would be refractory to ISGs that regulate virus envelopment or budding from the cell surface.

Human IFIT proteins include IFIT1, IFIT2, IFIT3 and IFIT5 (ISG56, ISG54, ISG60, and ISG58, respectively). IFIT expression is strongly induced upon viral infection (39, 40) and IFIT proteins limit the replication of multiple RNA viruses. IFIT1 was one of the first proteins shown to bind specifically to viral RNA (41) and further studies have shown that binding with IFIT3 increases its stability and specificity (34). In addition to direct-acting antiviral mechanisms involving the inhibition of translation, IFIT3 also has an indirect antiviral mechanism through its involvement in the RIG-I signaling pathway, where it functions as a molecular bridge between MAVS and TBK1 (18). This was described in the context of RNA virus sensing, but not expected to relate to DNA viruses which are sensed through the cGAS-STING axis. We demonstrated that IFIT3, but not IFIT1 or IFIT2, significantly inhibits Ad immediate gene expression. We previously demonstrated that type I and II IFN signaling repress HAdV-C5 immediate early gene expression and viral DNA replication in normal human cells (3), the same phenotype observed in the current experiments with IFIT3 expression alone. We found, however, that IFIT3 is not required for type I or II IFN inhibition of Ad gene expression and DNA replication (Fig. 2). IFIT3 also did not affect early stages of Ad infection leading to nuclear import of the viral genome (Fig. 3). Thus, we postulate that IFIT3 does not have direct-acting antiviral activity against Ad.

**FIG 9:**
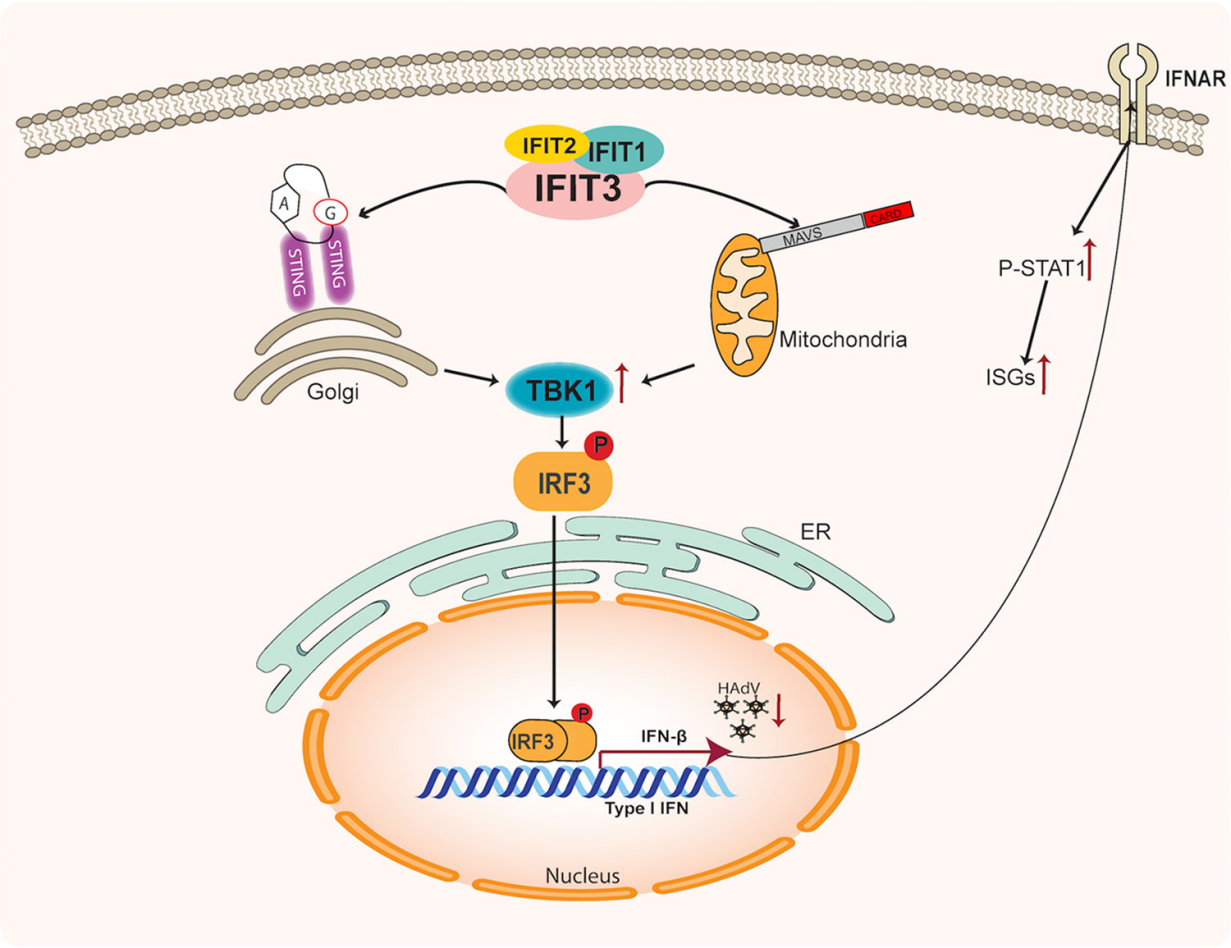
Schematic diagram of IFIT3 as an adaptor protein in innate immunity. Expression of IFIT3 in HDF cells leads to increased expression of IFIT1 and IFIT2, which forms a complex that activates the STING and mitochondrial antiviral signaling (MAVS) pathways. This leads to phosphorylation of TBK1 which, in turn, phosphorylates and activates IRF3. Phosphorylated IRF3 dimerizes and translocates to the nucleus where it activates interferon (IFN)β gene expression. IFNβ is secreted, binds to the IFNAR, and activates canonical IFN signaling and the induction of interferon-stimulated genes (ISG) expression. One or more ISGs block Ad E1A immediate early gene expression and viral DNA replication. A:G represents cGAMP, an activator of STING at the Golgi; MAVS association with mitochondria is shown.

Instead, our results demonstrate that IFIT3 acts indirectly to inhibit Ad by stimulating IFN signaling (Fig. 9). Our results are consistent with a model whereby a complex containing IFIT1, IFIT2 and IFIT3 stimulate TBK1 phosphorylation using both STING and MAVS activities. Activated TBK1 induces IRF3 phosphorylation and the induction of IFNβ gene expression. IFNβ, in turn, activates canonical IFN signaling with the induction of ISG expression. One or more ISG products inhibit Ad immediate gene expression and viral DNA replication. These results demonstrate that the expression of a single ISG, IFIT3, activates IFN signaling and establishes a cellular antiviral state independent of viral PAMPs. IFIT3 was previously shown to serve as an adaptor that bridges MAVS and TBK1 at the mitochondria to potentiate antiviral signaling (18), an effect that required RNA virus infection. In our experiments, cGAS depletion did not alter the effect of IFIT3 on IFN signaling or Ad replication and viral gene expression (Fig. 5). Further, IFIT3 stimulated IFN signaling independent of Ad infection, thus viral DNA sensing was not involved in IFIT3 activity. We conclude that IFIT3 functions downstream of cGAS and upstream or in conjunction with STING and MAVS. cGAS/STING and RIG-I/MAVS pathways are involved in cytoplasmic sensing of DNA and RNA PAMPs, respectively (33). To our knowledge, this is the first demonstration of direct cross talk between the STING and MAVS pathways, although it was previously shown that cGAS has antiviral activity against RNA viruses *in vitro* and *in vivo* (38).

IFIT proteins have been shown to form homodimers and heterodimers which enhances their functions (34). We demonstrated that IFIT3 induced the expression of IFIT1 and IFIT2, consistent with the activation of IFN signaling, and suggesting a potential role of these proteins in the IFIT3 response. In contrast to IFIT3, the individual expression of IFIT1 and IFIT2 expression did not inhibit E1A expression or Ad replication (Fig. 7). We do not understand why individual expression of IFIT3, but not IFIT1 or IFIT2, promotes this antiviral process. IFIT3 may be required to nucleate a functional IFIT protein complex. Poly-IC induces the expression of IFIT proteins via TLR3 signaling and knockdown of IFIT1, IFIT2 or IFIT3 inhibited the induction of phosphorylated STAT1 (42) consistent with results in our assays.

Ectopic expression of IFIT3 restricts the infection of multiple viruses, including porcine reproductive and respiratory syndrome virus (PRRSV), swine influenza virus (SIV), herpes simplex virus-1 (HSV-1), and Kaposi’s sarcoma herpesvirus (KSHV) (43–45). While the mechanism(s) by which IFIT3 restricts replication of the DNA viruses HSV-1 and KSHV is not known, the published results are consistent with activation of IFN signaling seen here and ISG-mediated viral restriction. IFIT3 was shown to enhance IFN-β promoter activity in response to poly-IC stimulation (44, 45) and promoted cell survival (46). Direct binding of IFIT3 with RNA has not been described; instead IFIT3 is thought to exert its antiviral effect indirectly by binding to other IFIT proteins or host defense molecules (41, 47, 48).

We previously demonstrated that type I and II IFNs inhibit the replication of divergent human Ads via an evolutionary conserved E2F binding site in the immediate early E1A gene transcriptional enhancer region (3). This interaction downregulates viral replication and infectious virus production ∼100-fold. One possible interpretation of this observation is that Ads use IFN signaling to suppress viral replication in order to establish and maintain persistent and latent viral infections (3). HAdV-C5 establishes a state of persistent viral infection in HDF cells in the presence of IFNα or IFNγ that can be maintained for months without the loss of cell viability. Withdrawal of IFN reactivates lytic infection and an Ad5 mutant virus that is refractory to the effects of IFNs is unable to establish persistent infection in this assay (3). It would be interesting to determine if HAdV-C5 can establish a persistent infection in HDF cells utilizing IFNs in the absence of IFIT3. In summary, we conclude that IFIT3 expression is sufficient to induce IFN signaling independent of viral PAMPs and in coordination with cytoplasmic pathways associated with both RNA and DNA virus recognition. We believe that Ads may utilize IFIT3 to promote persistent infection in a feed-forward loop to maintain IFN signaling over time.

## MATERIALS AND METHODS

### Cell culture and viruses.

A549 cells (ATCC), 293FT cells (Life Technologies), and normal human diploid fibroblasts immortalized by the expression of human telomerase (HDF-TERT)(23) were maintained in Dulbecco’s Modified Eagle’s Medium (DMEM) with 10% fetal bovine serum (FBS). All cell growth media were supplemented with 100 μg/ml penicillin and streptomycin. An Ad5-EGFP virus was created by the replacement of the E4ORF3 protein reading frame with EGFP coding sequences and was used for the initial ISG screen. Specifically, HAdV-C5 nt 34,352-34,704 (E4ORF3) were deleted and the EGFP reading frame inserted in the orientation for expression from the natural E4 promoter. Details of cloning manipulations are available upon request. Wild type Adenovirus-5 (HAdV-C5) was used for all subsequent experiments. Virus infections were performed for 1 h at 37°C at the multiplicities of infection (MOI) described in the figure legends followed by removal of virus and replacement with fresh medium. For A549 cells, an MOI of 5 virus particles/cell (P/cell) results in infection of ∼50% of the population. For HDF cells, an MOI of 200 P/cell results in infection of ∼one third of the population and an MOI of 1000 P/cells results in infection of ∼90% of the population.

### High-throughput microscopy screening of an ISG library.

An ISG library in the lentivirus expression vector pSCRPSY, and co-expressing RFP as a transduction control and puromycin resistance as a selection marker, was previously described (19). A549 cells in 96-well plates were first transduced with the individual Lentivirus library ISG clones and placed under puromycin selection, and then infected with Ad5-green fluorescent protein (GFP) at a low MOI (<1% GFP-positive cells at 24 hpi). Lentiviral transduction was performed in duplicate plates for each ISG, where one sample was fixed after one round of replication (24 hpi), defining the number of initial producer cells, and the other after several rounds of replication (60 hpi). “Spread ratio” was calculated by dividing the Ad5-EGFP-infected cells at 60 hpi by those at 24 hpi for each individual ISG over empty vector-transduced cells. This yielded results that were stable over a wide range of Lentivirus transduction efficiencies.

### Lentivirus production and transduction.

Lentivirus vectors expressing IFIT1, IFIT2, and IFIT3 were produced by transfection of 293FT cells with pSCRISPY vectors containing individual IFIT3 coding sequences (19), along with plasmids pLP1, pLP2, and pLP/VSV-G (Invitrogen). Culture supernatants were harvested 3–4 days after transfection, filtered using Millex-HV filters (Millipore), and stored at −80°C. HDF cells were transduced with pSCRISPY Lentivirus vectors and placed under puromycin selection 48 h after transduction. Pools of puromycin-resistant cells were used and IFIT protein expression was confirmed by Western blotting. pLenti-CRISPR-v2 vectors containing puromycin or blasticidin-resistance genes were from AddGene and were engineered to express 20 nt targeting sequences for IFIT1 (AGAGATCGCATACCCAGCGC), IFIT2 (AGAACGCCATTGACCCTCTG), IFIT3 (AAAATTTGGCTGCACTGCGG), JAK1 (TCCCATACCTCATCCGGTAG), TBK1 (GAAGAACCTTCTAATGCCTA), STING (AGAGCACACTCTCCGGTAC), cGAS (CGCATCCCTCCGTACGAGAA), and MAVS (GAGGGCTGCCAGGTCAGAGG). Individual colonies were isolated following Lenti-CRISPR transduction of HDF cells and screened by Western blotting for gene knockout. For gene knockout and IFIT3 overexpression, HDF cells were cotransduced with Lenti-SCRISPY-IFIT3 and Lenti-CRISPR vectors, selected using puromycin and blasticidin, and individual colonies isolated and screened by Western blotting for IFIT3 expression and gene knockout.

### Viral replication assay and RT-qPCR.

HDF and HDF-IFIT3 cells were infected with HAdV-C5 for 1 h at 37°C at the multiplicities of infection indicated in the text and figure legends. The infection inoculum was removed, and fresh medium added. Total cellular DNA was purified at 6 h and 48 h postinfection (hpi) using a Qiagen DNeasy blood and tissue kit. Both viral and cellular genome copy numbers were determined by qPCR using primer pairs that recognize either the Ad5 genome or cellular glyceraldehyde-3- phosphate dehydrogenase (GAPDH) gene using DyNAmo HS SYBR green qPCR Kit (Thermo). After normalizing the viral DNA copy numbers to GAPDH, the fold-increase in viral copy numbers was calculated by normalizing the amount of DNA present at 48 h to the amount present at 5 h. With A549 cells, total cellular DNA was harvested at 4 and 24 hpi. For RT-qPCR, cells were infected for 48 h and total cellular RNA was isolated using a Qiagen RNeasy kit. Equal amounts of total RNA were used to synthesize the first strand cDNA using a SuperScript II Reverse Transcriptase and oligo-dT primer (Life Technologies). Equals amount of cDNA were then subjected to qPCR using primer pairs that recognize individual ISG mRNAs and cellular GAPDH mRNA. ISG mRNA was normalized to the internal control GAPDH mRNA.

### Immunofluorescence.

HDF cells were seeded on glass coverslips and left untreated or treated with IFNα for 24 h. HDF-IFIT3 cells were not treated with IFN. At time points indicated in the figure legend, cells were washed, fixed with 4% (vol/vol) formaldehyde, and permeabilized with 0.5% Triton X-100 and washed. After blocking in 10% goat serum, coverslips were incubated with IFIT3 antibody (GeneTex, 112442, 1:300) and monoclonal antibodies against Ad DNA binding protein (A1-6 and B6-8 (49), 1:100 each) for 24 h at 4°C. The coverslips were washed, incubated with FITC-labeled anti-rabbit IgG and TRICT-labeled anti-mouse IgG (Amersham, 1:300) and DAPI for 1 h at room temperature. Coverslips were washed and mounted on glass slides using Immunomount (Shandon). Images were captured using Zeiss Axiovert 200M digital deconvolution microscope with Axiovision 4.8.2 SP3 software.

Viral DNA entry into the nucleus was determined using IF by infecting HDF and HDF-IFIT3 cells with HAdV-C5 at an MOI of 1000 particles/cell. At 7 hpi, cells were fixed using ice-cold methanol, washed and incubated with anti-VII primary antibody. Secondary antibody was added, and coverslips mounted on slides. Images were captured by structured illumination microscopy (N-SIM, Nikon) and images were reconstructed and analyzed using NIS-Elements software (Nikon).

### Western blot analysis.

Whole cell extracts were prepared by suspending cell pellets in SDS lysis buffer (50 mM Tris-HCl, pH 6.5, 2% SDS and 10% glycerol) and boiled for 10 min. Protein concentration was determined using Pierce BCA Protein assay kit. Equal amounts of proteins were resolved on SDS-PAGE gel and then transferred to a nitrocellulose membrane. The membranes were blocked in Tris-HCL-buffered-saline (TBS) buffer containing 3% BSA for 1 h at room temperature. Following the blocking, primary antibodies against protein were added (as indicated in figure legends and text) at 4°C overnight. Membranes were washed with TBS buffer containing 0.1% Tween 20 (TBS-T) and then incubated with IRDye 800CW-cojugated goat anti-rabbit antibody (926-32211, Li-COR, 1:5000) and IRDye 680RD- conjugated goat anti-mouse antibody (925-068071, Li-COR, 1:5000) for 1 h at room temperature. After three washes with TBS-T buffer, images were captured using the ODYSSEY CLx infrared imaging system (Li-COR). Alternatively, HRP- conjugated antibodies (Amersham) were also used in conjunction with ECL Western blotting (Millipore Immobilon) and images captured using a GE ImageQuant LAS 500. The following antibodies were used: IFIT1 and IFIT2 (ProteinTech Group, 23247-1-AP and 12604-1-AP, 1:1000), IFIT3 (GeneTex, 112442, 1:1000), total STAT1 (Cell Signaling Technology, 9172, 1:1000), phospho-STAT1 (Y101)(Cell Signaling Technology, 9167, 1:1000), total TBK1 (Cell Signaling Technology, 3504, 1:1000), phospho-TBK1 (S396) (Cell Signaling Technology, 5483, 1:1000), JAK1 (Cell Signaling Technology, 3344, 1:1000), STING (Cell Signaling Technology, 13647, 1:1000), cGAS, and MAVS (Cell Signaling Technology, 24930, 1:1000), Ad5 E1A (NeoMarkers, M73, MS-588-P, 1:1000), Ad5 Hexon (Abnova, MAB8757, 1:5000), and α-Tubulin (Sigma-Millipore, T5168, 1:10,000).

### Statistical analyses.

Statistical significance of the differences was calculated using Student's *t* test and is presented as mean +/- standard deviation. Each experiment was done in three replicates.
