# Supplementary figures and images for: Human IFIT3 Protein Induces Interferon Signaling and Inhibits Adenovirus Immediate Early Gene Expression
Source: mBio. 2021 Nov 2;12(6):e02829-21. doi: 10.1128/mBio.02829-21 (PMC8561380; doi:10.1128/mBio.02829-21)

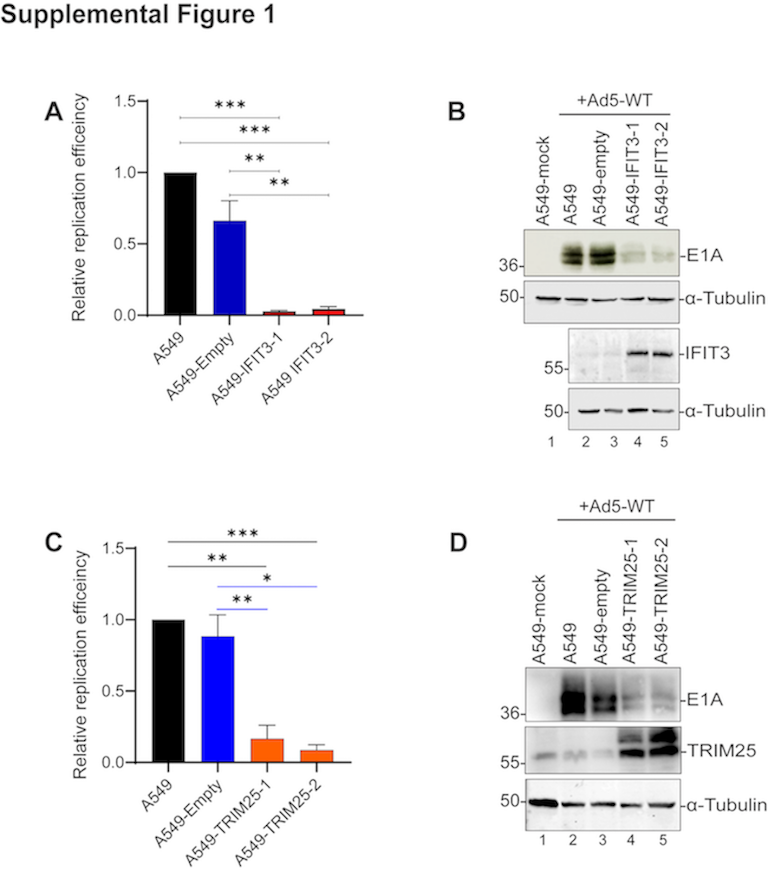

Supplement: FIG S1 [file mbio.02829-21-sf001.tif]

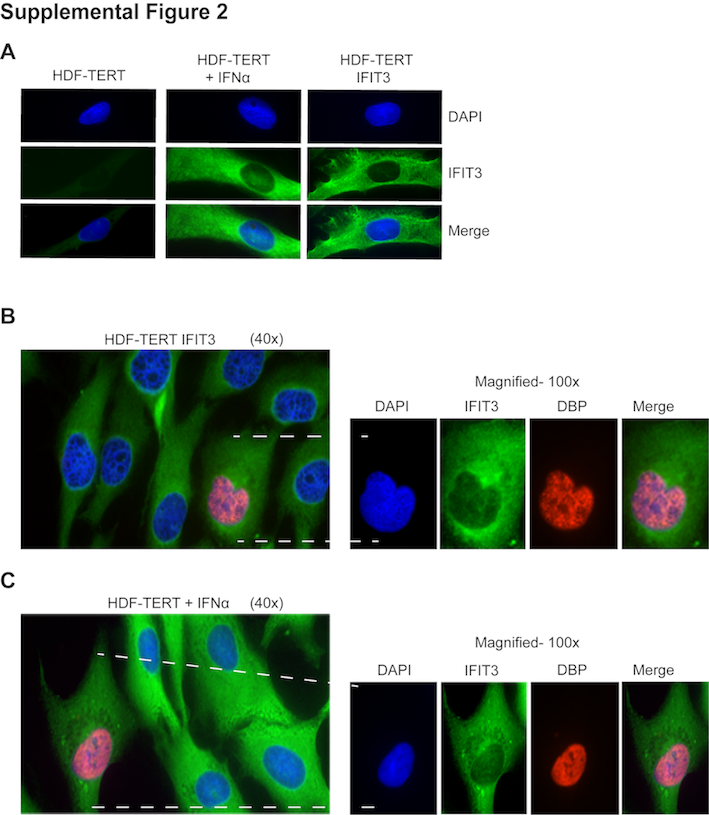

Supplement: FIG S2 [file mbio.02829-21-sf002.tif]

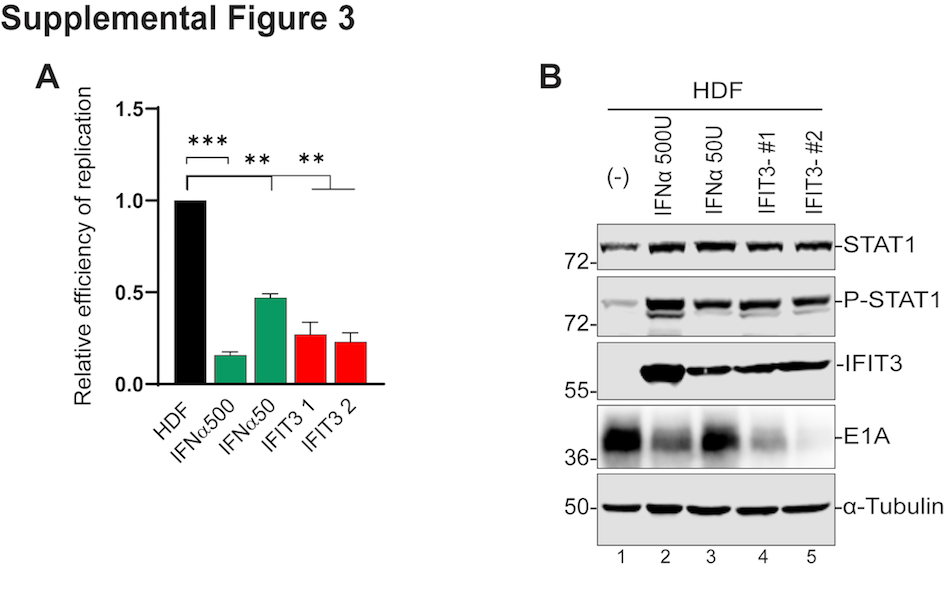

Supplement: FIG S3 [file mbio.02829-21-sf003.tif]

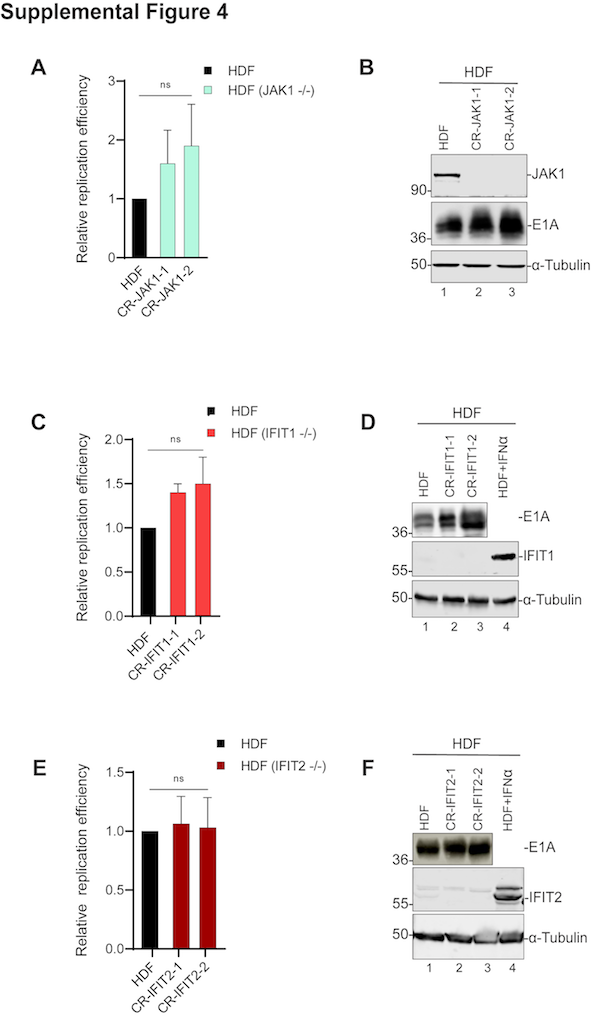

Supplement: FIG S4 [file mbio.02829-21-sf004.tif]

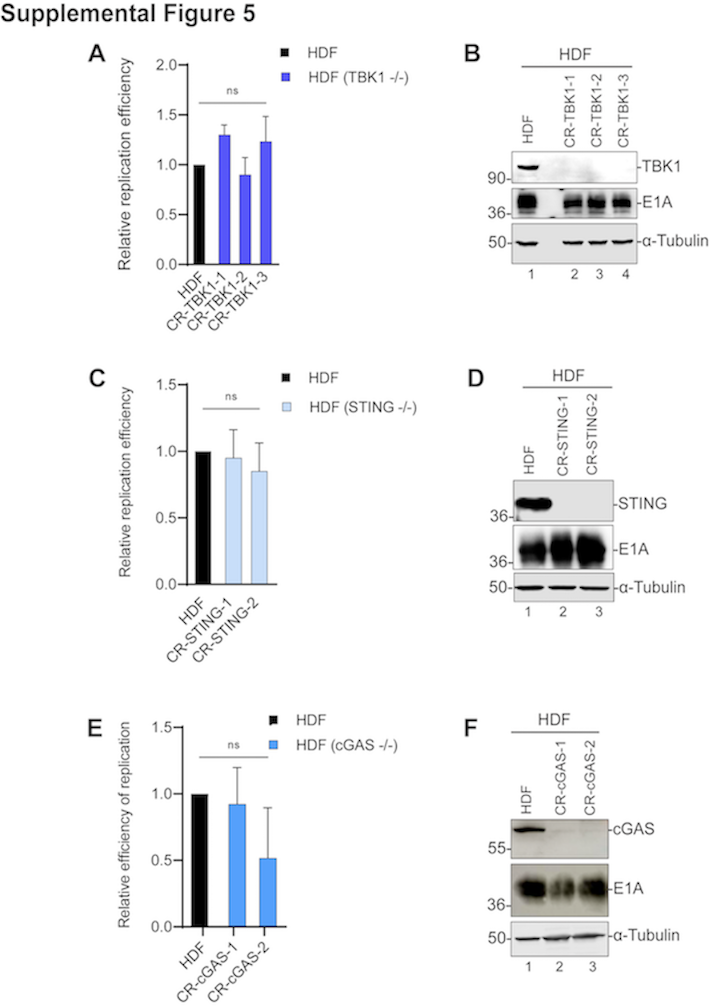

Supplement: FIG S5 [file mbio.02829-21-sf005.tif]

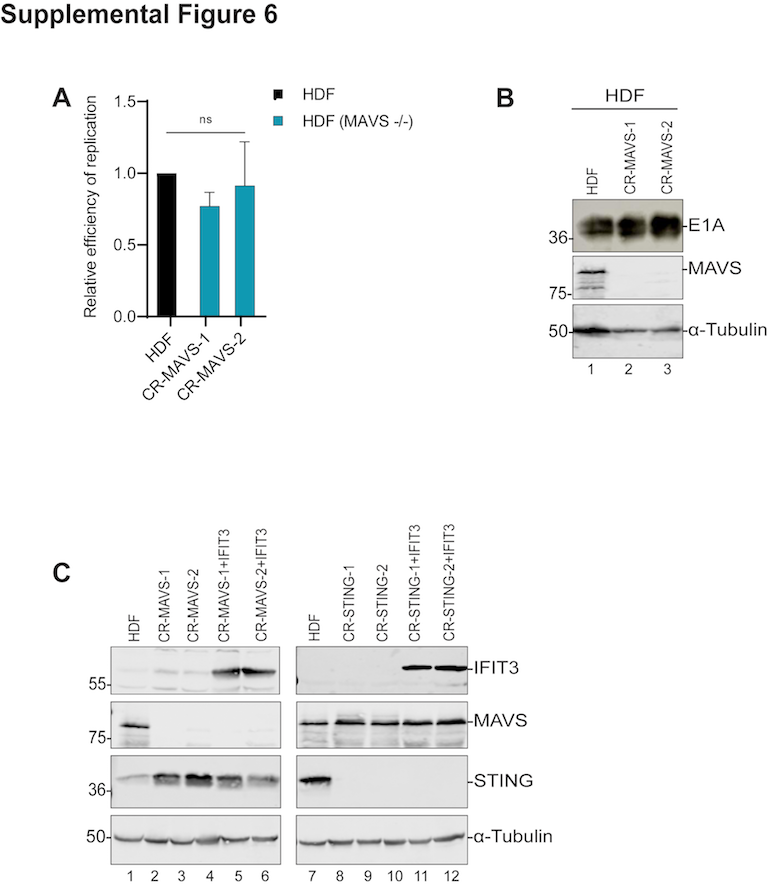

Supplement: FIG S6 [file mbio.02829-21-sf006.tif]
